# Supplementary material for: Effective field theories for interacting boundaries of 3D topological crystalline insulators through bosonisation
Source: Sci Rep. 2020 Dec 15;10:21998. doi: 10.1038/s41598-020-77966-3 (PMC7738686; doi:10.1038/s41598-020-77966-3)
Supplement: Supplementary file 1 — Supplementary Informations. [file 41598_2020_77966_MOESM1_ESM.pdf]

Supplementary information: Effective field theories for interacting boundaries of 3D topological crystalline insulators through bosonisation

Patricio Salgado-Rebolledo,[1,*] Giandomenico Palumbo,[2] and Jiannis K. Pachos[1]

[1] *School of Physics and Astronomy, University of Leeds, Leeds, LS2 9JT, United Kingdom*
[2] *Center for Nonlinear Phenomena and Complex Systems, Université Libre de Bruxelles, CP 231, Campus Plaine, B-1050 Brussels, Belgium*
(Dated: October 16, 2020)

Appendix A: Interpolating action

In this Appendix we consider a generalisation of the interpolating action method [29] to the case of an interacting Chern-Simons-Proca model of the form (25):

$$\mathcal{S}[a,b]=\int d^3x\left[-\frac{1}{2}a^\mu a_\mu-\frac{1}{2}b^\mu b_\mu+\frac{1}{2m_a}\epsilon^{\mu\nu\rho}a_\mu\partial_\nu a_\rho+\frac{1}{2m_b}\epsilon^{\mu\nu\rho}b_\mu\partial_\nu b_\rho+\frac{1}{m_i}\epsilon^{\mu\nu\rho}a_\mu\partial_\nu b_\rho\right], \tag{A1}$$

where here the constants m_a, m_b and m_i are considered as arbitrary. In order to do so, we introduce two gauge fields $\mathcal{A}_\mu$ and $\mathcal{B}_\mu$ and consider the following general form for an interpolating action

$$\begin{aligned}\mathcal{S}_I[a,b,\mathcal{A},\mathcal{B}]=\int d^3x\bigg[&-\frac{1}{2}a^\mu a_\mu-\frac{1}{2}b^\mu b_\mu+\lambda_1\epsilon^{\mu\nu\rho}a_\mu\partial_\nu\mathcal{A}_\rho+\lambda_2\epsilon^{\mu\nu\rho}b_\mu\partial_\nu\mathcal{B}_\rho\\&+\lambda_3\epsilon^{\mu\nu\rho}b_\mu\partial_\nu\mathcal{A}_\rho+\lambda_4\epsilon^{\mu\nu\rho}a_\mu\partial_\nu\mathcal{B}_\rho-\frac{m_\mathcal{A}}{2}\epsilon^{\mu\nu\rho}\mathcal{A}_\mu\partial_\nu\mathcal{A}_\rho-m_\mathcal{I}\epsilon^{\mu\nu\rho}\mathcal{A}_\mu\partial_\nu\mathcal{B}_\rho-\frac{m_\mathcal{B}}{2}\epsilon^{\mu\nu\rho}\mathcal{B}_\mu\partial_\nu\mathcal{B}_\rho\bigg]\end{aligned} \tag{A2}$$

together with the corresponding path integral

$$\mathcal{Z}_I=\int\mathcal{DADB}\mathcal{D}a\mathcal{D}b\,\exp\left\{i\int d^3x\mathcal{S}_I[a,b,\mathcal{A},\mathcal{B}]\right\}, \tag{A3}$$

Here we have defined the masses m_A, m_B and m_I, which are the coupling constants of the model, while λ_n ($n=1,2,3,4$) stands for a set of real constant coefficients that can be functions m_A, m_B and m_I.

By independently integrating out the pairs of fields a_μ, b_μ and $\mathcal{A}_\mu$, $\mathcal{B}_\mu$, the generating functional (A3) interpolates between the path integral associated to the action (A1) and an effective action for the fields $\mathcal{A}_\mu$ and $\mathcal{B}_\mu$, which means that up to normalisation factors,

$$\int\mathcal{D}a\mathcal{D}b\,\exp\{i\mathcal{S}[a,b]\}=\mathcal{Z}_I=\int\mathcal{DADB}\,\exp\{i\mathcal{S}^{\text{dual}}[\mathcal{A},\mathcal{B}]\}. \tag{A4}$$

One can show that the action $\mathcal{S}^{\text{dual}}[\mathcal{A},\mathcal{B}]$ is given by

$$\begin{aligned}S^{\text{dual}}_{\text{eff}}[\mathcal{A},\mathcal{B}]=\int d^3x\bigg[&-\frac{1}{4}(\lambda_1^2+\lambda_3^2)\mathcal{F}_{\mu\nu}\mathcal{F}^{\mu\nu}-\frac{1}{4}(\lambda_2^2+\lambda_4^2)\mathcal{G}_{\mu\nu}\mathcal{G}^{\mu\nu}-\frac{1}{2}(\lambda_1\lambda_4+\lambda_2\lambda_3)\mathcal{F}_{\mu\nu}\mathcal{G}^{\mu\nu}\\&-\frac{m_\mathcal{A}}{2}\epsilon^{\mu\nu\rho}\mathcal{A}_\mu\partial_\nu\mathcal{A}_\rho+m_\mathcal{I}\epsilon^{\mu\nu\rho}\mathcal{A}_\mu\partial_\nu\mathcal{B}_\rho-\frac{m_\mathcal{B}}{2}\epsilon^{\mu\nu\rho}\mathcal{B}_\mu\partial_\nu\mathcal{B}_\rho\bigg].\end{aligned} \tag{A5}$$

---

*Electronic address: psalgadoreb@gmail.com

where we have defined the field strengths $\mathcal{F}_{\mu\nu} = \partial_\mu \mathcal{A}_\nu - \partial_\nu \mathcal{A}_\mu$, $\mathcal{G}_{\mu\nu} = \partial_\mu \mathcal{B}_\nu - \partial_\nu \mathcal{B}_\mu$, and the constants $m_\mathcal{A}$, $m_\mathcal{B}$, $m_\mathcal{I}$ are determined by the relations

$$
\begin{aligned}
m_a &= \frac{m_\mathcal{A} m_\mathcal{B} - m_\mathcal{I}^2}{\lambda_4^2 m_\mathcal{A} + \lambda_1^2 m_\mathcal{B} + 2\lambda_1\lambda_4 m_\mathcal{I}}, \\
m_b &= \frac{m_\mathcal{A} m_\mathcal{B} - m_\mathcal{I}^2}{\lambda_2^2 m_\mathcal{A} + \lambda_3^2 m_\mathcal{B} + 2\lambda_2\lambda_3 m_\mathcal{I}}, \\
m_i &= \frac{m_\mathcal{A} m_\mathcal{B} - m_\mathcal{I}^2}{\lambda_4\lambda_2 m_\mathcal{A} + \lambda_1\lambda_3 m_\mathcal{B} + (\lambda_1\lambda_2 + \lambda_3\lambda_4) m_\mathcal{I}}.
\end{aligned}
\tag{A6}
$$

Models with this type of interaction in the for the gauge fields and their corresponding curvatures have been considered in effective descriptions of incompressible quantum fluids and Josephson junction arrays [56–58]. Different choices for the coefficients λ_n are translated into different choices of gauge fields $\mathcal{A}_\mu$ and $\mathcal{B}_\mu$. The fields A_μ and B_μ considered in (26) correspond to the case $\lambda_3 = \lambda_4 = 0$, $\lambda_1 = \lambda_2 = 1$. In this case one can solve (A6) to find

$$
\begin{aligned}
m_\mathcal{A} &= \frac{m_a m_i^2}{m_i^2 - m_a m_b}, \\
m_\mathcal{B} &= \frac{m_b m_i^2}{m_i^2 - m_a m_b}, \\
m_\mathcal{I} &= \frac{m_a m_b m_i}{m_i^2 - m_a m_b}.
\end{aligned}
\tag{A7}
$$

Thus, using the values $m_a = 4\pi s_m/(e_\psi^2 + e_\chi^2)$, $m_b = 4\pi s_m/\alpha^2$ and $m_i = 4\pi s_m/e_\psi \alpha$ given in (25), one finds (28).
